# Influence of upwelling on coral reef benthic communities: a systematic review and meta-analysis

**DOI:** 10.1098/rspb.2023.0023

**Published:** 2023-03-29

**Authors:** Danielle L. Spring, Gareth J. Williams

**Affiliations:** School of Ocean Sciences, Bangor University, Menai Bridge, Anglesey LL59 5AB, UK

**Keywords:** benthic competition, nutrient flux, temperature variation, internal waves, environmental drivers, evidence synthesis

## Abstract

Highly competitive coral reef benthic communities are acutely sensitive to changes in environmental parameters such as temperature and nutrient concentrations. Physical oceanographic processes that induce upwelling therefore act as drivers of community structure on tropical reefs. How upwelling impacts coral communities, however, is not fully understood; upwelling may provide a natural buffer against climate impacts and could potentially enhance the efficacy of spatial management and reef conservation efforts. This study employed a systematic review to assess existing literature linking upwelling with reef community structure, and a meta-analysis to quantify upwelling impact on the percentage cover of coral reef benthic groups. We show that upwelling has context-dependant effects on the cover of hard coral and fleshy macroalgae, with effect size and direction varying with depth, region and remoteness. Fleshy macroalgae were found to increase by 110% on inhabited reefs yet decrease by 56% around one well-studied remote island in response to upwelling. Hard coral cover was not significantly impacted by upwelling on inhabited reefs but increased by 150% when direct local human pressures were absent. By synthesizing existing evidence, this review facilitates adaptive and nuanced reef management which considers the influence of upwelling on reef assemblages.

## 1. Introduction

Tropical coral reefs are dynamic socioecological systems that support the health and wellbeing of hundreds of millions of people [1]. Over the past few decades, coral reefs worldwide have undergone unprecedented change driven by cross-scale human impacts [2,3]. These include local drivers such as overfishing and land-based pollution and global climate change-induced ocean warming events that trigger disease outbreaks [4], mass coral bleaching and mortality [5]. While governments strive to reduce greenhouse gas emissions and slow the rate of ocean warming, local resource managers are tasked with safeguarding coral reefs and the ecosystem services they provide to humanity. These efforts are necessarily undertaken against a backdrop of environmental variability that constrains reef ecosystem structure and function [6,7] and in doing so sets a natural bound on what resource managers can achieve. They therefore require evidence-based guidance on how local environmental context might constrain, support or hinder their conservation efforts and goals.

Reef-builders on tropical coral reefs including calcifying (scleractinian) corals and crustose coralline algae (CCA) compete for space on the reef floor with non-accreting fleshy organisms such as turf algae and larger seaweeds. The outcomes of these competitive interactions are affected by changes in environmental parameters driven by biogeochemical and physical oceanographic processes [8–10]. Upwelling and the breaking of deep-water internal waves cause nutrient-rich deep water to propagate into the shallows [11]. Coastal upwelling is caused by two primary mechanisms: the movement of surface waters driven by wind energy moving along or away from shore, and when an island mass blocks the trajectory of current-driven water movement, causing deeper waters to shoal [11,12]. In stratified waters, internal waves can form at the interface between two water masses with different densities, in much the same way that a surface wave propagates between the boundary of seawater and the atmosphere [13]. Generated by strong tidal flows interacting with rough bottom topography [14], internal waves cause ocean mixing which in turn transports deep, cooler and nutrient-rich waters toward the surface [15]. Wind-driven upwelling and the propagation of internal waves are exclusive processes with different mechanisms; here, ‘upwelling’ refers to all processes driving cool pulses of deep water onto shallow coral reefs.

Upwelling can have variable effects on coral reef communities [16,17]. As mixotrophic organisms, reef-building corals obtain their energy and nutritional needs through a combination of autotrophy in symbiosis with the photosynthetic microalgae found within the coral tissue, and heterotrophic feeding by the coral animal through capture of particles within the water column [18,19]. This strategy of trophic plasticity underpins the success of coral reefs, supporting inherent flexibility and adaptation of corals that allows reefs to thrive under variable environmental conditions [18,20]. In otherwise nutrient-poor waters, increased nutrient supply may act in favour of coral productivity and growth by providing an additional energy source to supplement autotrophic feeding [21]. Upwelling does not always promote coral productivity, however; cold pulses of upwelled water can have detrimental effects on scleractinian corals [22] by reducing water temperatures below the lower limit of the coral's thermal threshold [10,23–25]. In tandem with less favourable temperatures for corals, upwelling can favour algal species which are able to efficiently and opportunistically utilize the influx of biologically available nutrients brought up from deeper waters [23,26,27].

The varied responses of benthic communities to biophysical drivers may be altered or entirely reversed in areas subject to direct local human impacts [28]. Where background nutrient concentrations are high due to terrestrial run-off caused by poor watershed management, or herbivorous fish populations that control algal growth are removed by intensive fishing, the somewhat predictable patterns in benthic community structure on isolated reefs are disrupted [28,29]. Exactly how upwelling shapes competitive interactions of benthic groups on coral reefs is unclear, and likely dependent on the spatial and temporal variability of co-occurring environmental and anthropogenic forces. The variation in study results linking upwelling to reef community structure have produced a contradictory array of conclusions, with some studies reporting upwelling resulting in algal dominance [23,26,27] and others finding coral proliferation [29,30].

Given the concerning global trajectory of coral reefs [31], active management is necessary to secure a future for reef ecosystems. Because human intervention must happen in the context of natural environmental variability, such variability should be incorporated into adaptive management plans. By focusing conservation strategies on supporting reefs' natural resilience and integrating active human intervention with natural mitigation of reef degradation, positive outcomes for maintaining coral reefs may become more likely. This is particularly true when we consider the finite financial resources available to support conservation efforts [32]. Since a warming climate poses the greatest threat to coral survivability [2,33,34], environmental phenomena that reduce temperature to within the thermal tolerance range of corals may confer resistance to coral bleaching and subsequent mortality [32]. Upwelling may create local scale pockets of refugia from thermal stress and may therefore be sites best placed to focus conservation efforts [14,35]. Given that patterns of upwelling are likely to change in concert with global climate change, understanding biological responses to upwelling dynamics is necessary for predicting future conditions of reef communities.

This review seeks to systematically assess the existing body of evidence relating upwelling to benthic community structure on coral reefs and to provide a policy-neutral summary of existing evidence. Systematic reviews linking reef health with anthropogenic stressors including pollution [36], sediment exposure [37], chemical pollutants [38] and anthropogenic nutrient enrichment [39] have provided valuable overview analysis of the state of evidence. Such broad evidence synthesis allows policy makers and reef managers to make informed, evidence-based decisions founded in robust science. Although upwelling affects coral reefs throughout the ocean, no such review exists which comprehensively synthesizes the research linking changes in environmental parameters driven by upwelling with associated impacts on coral reef benthic communities.

The results of this study were anticipated to highlight the variability of upwelling impacts on reef communities. The hypotheses were, firstly, that benthic groups would exhibit differential responses to upwelling dependant on their functional morphology; non-calcifying organisms such as turf and fleshy macroalgae were expected to increase in abundance due to their ability to opportunistically utilize nutrient influx [23,26]. Secondly, responses of benthic communities were hypothesized to differ between remote reefs and those close to human population centres, as local anthropogenic stressors are demonstrated to disrupt natural biophysical relationships [8,28,40]. Hard coral cover was expected to respond positively to upwelling where local anthropogenic stressors are absent, as the potential nutritional benefits of upwelling to corals are likely to be overshadowed by the presence of human populations. By synthesizing the existing body of evidence, this review will facilitate enhanced understanding of reef community responses to upwelling, supporting resource managers and decision makers in creating nuanced and informed reef management and conservation policy.

## 2. Methods

### (a) Study design

This study employed a systematic review and meta-analysis to assess the impact of upwelling on the relative dominance of benthic groups on coral reefs, following guidance set out by Pullin & Stewart [41], the Collaboration for Environmental Evidence [42] and the Preferred Reporting Items for Systematic Reviews and Meta-Analysis (PRISMA) [43]. Key elements of the review question can be viewed using the PECO format (CEE Guidelines v.5.0., 2018 [44]):

Population—coral reef benthic communities on shallow (≤30 m) tropical reefs (between 30° N and 30° S).
Exposure—cold pulses of deep water driven by upwelling.
Comparator—comparable sites not subject to the impact of cold pulses driven by upwelling, or sites that are seasonally subject to upwelling (comparing upwelling and non-upwelling seasons).
Outcome—changes in the percentage cover of benthic groups.

Coral reef benthic communities were categorized into the following 6 groups, following Williams *et al*. [45]: hard coral, fleshy macroalgae, CCA, turf algae (including filamentous cyanobacteria), other calcifying macroalgae (e.g. *Halimeda* and *Peyssonnelia*) and soft coral. These were further defined by functional group, either calcifying (hard coral, CCA, calcifying macroalgae) or fleshy (fleshy macroalgae, turf algae, soft coral) organisms. The metric used to assess the impact of upwelling on the relative dominance of groups was percentage cover, as this was the predominant unit of measurement for assessing benthic community structure within the literature.

To further investigate the nuances of upwelling impacts, this review sought to decipher variability in impacts to benthic groups dependant on remoteness (distance from human population centres), depth, magnitude of the cold pulse (measured as the resulting temperature drop in °C) and geographic location.

### (b) Literature search and screening

Scoping of a search strategy was undertaken using the systematic review package litsearchr [46] in R (www.r-project.org). Terms generated in litsearchr were refined and tested on an iterative basis in Web of Science (electronic supplementary material, table S1) against a benchmark list of 10 key papers known to be highly relevant to the subject (electronic supplementary material, table S2). Following PRISMA guidelines [43], results retrieved at each stage of the search were recorded (see electronic supplementary material, figure S1 for PRISMA flow diagram). The final search was undertaken on 23/06/2022, capturing all key benchmark papers: *cora*l* OR reef* AND *upwelling* OR ‘internal wave*’ OR cooling-hour* OR ‘cooling hour*’ OR ‘cold pulse*’ AND *abundance* OR assemblage* OR alga* OR carbon* OR communit* OR *diversity* OR dynamic* OR dominan* OR ecosystem* OR *growth* OR nutrient* OR pattern* OR rate* OR benth* OR composition* OR develop* OR distribut* OR *production* OR response* OR seascape* OR *spatial* OR structur* OR zon* OR *trophic* OR varia* OR regime* OR ‘physical driver*’.

Web of Science (Core Collection database) and Scopus were used to search for literature, with a supplementary search of the first 200 results in Google Scholar to account for grey literature [47] (electronic supplementary material, table S3). Eligibility criteria were determined *a priori* (electronic supplementary material, table S4)*;* to be included in the review, studies must have undertaken comparative assessment of benthic communities under upwelling and non-upwelling conditions on coral reefs between 30° N and 30° S at depths of ≤30 m. This comparison could be either spatial (comparative sites, one of which is subjected to upwelling and the other not) or temporal (consideration of the same site during seasonal upwelling and during non-upwelling season). No temporal limitation was placed upon the search for literature.

Papers were screened at title (*n* = 1441) and abstract (*n* = 453) level and imported into the reference management software Mendeley for full-text screening. Ultimately, 17 studies met the inclusion criteria for use in the meta-analysis (electronic supplementary material, table S5): 16 peer-reviewed papers and a PhD thesis [10,14,23–27,29,30,48–54].

### (c) Data coding strategy

The following meta-data were extracted from 17 studies:

— Bibliographic information (study identifier, bibliographic source, title, author, journal, year, DOI, language and publication type).
— General description of the study (country, region, latitude and longitude coordinates, specific study location).
— Population description (benthic group, functional group).

Studies were also coded into predefined categories for the following variables:

— Functional morphology (calcifying or fleshy).
— Depth category of benthic cover assessment (shallow 0–10 m, moderate 11–20 m, deep 21–30 m, where case studies were categorized based on the majority of sampling effort – i.e. where target benthic sampling depth was 6–12 m, the study was classified as ‘shallow’).
— Geographic location.
— Remoteness: deemed ‘remote’ if local population is less than 50 people and greater than 100 km from human population centres, following Williams *et al*. [55].
— Whether benthic cover comparison featured spatial or temporal (seasonal) upwelling.

Quantitative data extracted for use in the meta-analysis included: mean percentage cover of benthic groups, standard deviation of percentage cover, number of independent study replicates, and mean temperature recorded during comparative upwelling and non-upwelling (°C).

### (d) Data extraction

Data were extracted directly from article texts, tables and figures (using Automeris WebPlotDigitizer v. 4.5) and by requesting data directly from authors where it was not readily available in the publication. A total of 188 case studies (multiple independent studies produced from a single paper, for example, where multiple benthic groups were assessed at numerous comparable locations) were extracted from 17 papers (see Data Coding and Meta-Data Extraction in Dryad Data Repository [56]).

Studies were critically appraised to assess for validity before being included in the meta-analysis. Studies were categorized as having ‘high’ or ‘low’ validity based on control matching of study and control conditions, habitat comparability between study and control sites, study replication and length and presence of confounding factors that may modify the effect of upwelling (i.e. proximity to aquaculture facilities).

### (e) Data analysis

A weighted meta-analysis was conducted on studies retrieved through the process of systematic review to assess the impact of upwelling on the percentage cover of benthic groups on coral reefs. Changes in the relative dominance of benthic groups were assessed by calculating a response ratio to quantify the proportionate change in the mean percentage cover of groups between comparative upwelling and non-upwelling conditions [57]. The natural logarithm of the response ratio, ln(RR), was calculated using the following equation:

$$\mathrm{lnRR}=\ln\left(\begin{array}{c} \bar{X}e\\ \bar{X}c \end{array}\right)=\ln(\bar{X}e)-\ln(\bar{X}c),$$

where X̄*e* is the mean percentage cover during upwelling and X̄*c* is the mean percentage cover during non-upwelling. A negative value indicates a reduction in percentage cover during upwelling and a positive value indicates an increase in percentage cover, comparative to non-upwelling.

Potential publication bias, or the likelihood of studies with significant or positive results to reach publication, was assessed using Egger's test for asymmetry together with a funnel plot of lnRR with standard error [58], which did not identify significant publication bias across studies (*R*^2^ = 0.093, *p* = 0.545), (see electronic supplementary material, figure S2). An *I^2^* statistic was generated to describe the proportion of variation in effect sizes across studies that is due to heterogeneity rather than chance [59]; a Cochran's *Q*-value was used to show the level and significance of heterogeneity [60]. Heterogeneity of effect sizes with associated *p*-values and *I*^2^ values for all models can be viewed in electronic supplementary material, table S6.

Having calculated effect size for each study (*k* = 180, where *k* represents independent case studies considered), a random effects model was used to assess the overall impact of upwelling on cover of benthic groups using the ‘rma.mv’ function within the ‘metafor’ package in R [61]. A random/mixed effects model was chosen as effect sizes were anticipated to vary from study to study and between different groups [62]. Publication ID was included as a random effect in all models to account for lack of independence of effects from the same study.

The model showed significant heterogeneity in effects between case studies. Therefore, subgroup analysis of benthic groups split by functional morphology, location, proximity to people and sampling depth was undertaken. Meta-regressions to investigate the impact of upwelling magnitude on benthic cover were conducted using mixed effects models. Magnitude of upwelling was quantified as the mean °C drop experienced during upwelling compared to non-upwelling.

## 3. Results

### (a) Summary findings and distribution of studies

In total, 180 case studies were analysed from 15 papers, spanning 5 countries, namely Colombia (*n* = 60), Costa Rica (*n* = 12), Panama (*n* = 24), Thailand (*n* *=* 13) and the United States Minor Outlying Islands (*n* *=* 71). Eight further case studies were not included in the final meta-analysis; three due to low comparability of upwelling and non-upwelling sites, (the Philippines, *n* *=* 1, and Taiwan, *n* *=* 2), and 5 due to zero percentage cover values, as lnRR cannot be applied to values of zero (United States Minor Outlying Islands, *n* = 5). Zero percentage cover values were explored for relevance and deemed appropriate for removal (see electronic supplementary material including figure S3 for exploratory analysis of these case studies). All studies were published between 2002 and 2022, with benthic community assessment spanning 1994–2019. Study effort was clustered around four geographic zones: Southeast Asia (*n* = 16), Pacific Central America (*n* = 36), the Caribbean (*n* = 60) and the Equatorial Pacific, specifically Jarvis Island (*n* = 76). See electronic supplementary material, figure S4 for map of study locations.

### (b) Effect of upwelling on benthic groups

**Figure 1.**
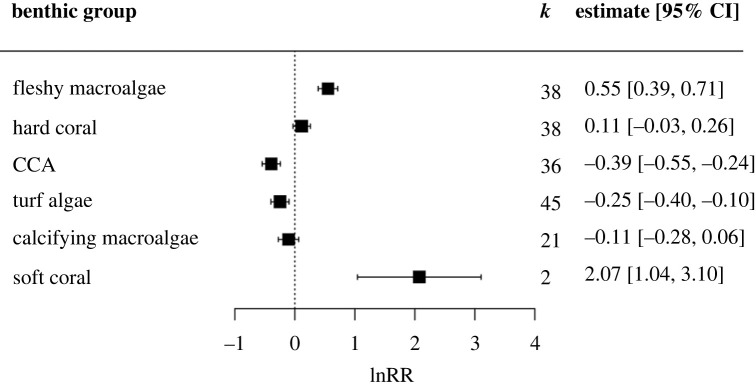
The lnRR (natural logarithm of response ratio) showing the effect of upwelling on the percentage cover of benthic groups on coral reefs. Boxes and error bars represent lnRR pooled effect sizes and 95% confidence intervals; values falling to the left of the dotted line indicate a negative effect of upwelling on the percentage cover of benthic groups, and to the right a positive effect. *k* represents the number of case studies that consider each benthic group included in the meta-analysis.

A multivariate mixed effects model with benthic group as a moderator showed that the percentage cover of fleshy macroalgae, CCA, turf algae and soft coral was significantly different during upwelling compared to non-upwelling (figure 1). A pooled significant effect of upwelling was not detected for other calcifying macroalgae or hard coral. Upwelling had a significant positive effect on the percentage cover of fleshy macroalgae and soft coral, increasing mean percentage cover by 73 and 692%, respectively. Given that only 2 studies considered the impact of upwelling on soft coral, this result cannot be considered conclusive, but may be indicative of actual effect. Upwelling had a significant negative effect on CCA, resulting in a 32% decrease in CCA cover. Similarly, the percentage cover of turf algae decreased by 22% with upwelling compared to non-upwelling. Effect size and direction varied across studies for all groups. Hard coral cover exhibited an almost even distribution of positive and negative effects with upwelling across studies (electronic supplementary material, figure S5).

The percentage of variability in effect sizes across studies attributed to heterogeneity rather than sampling error was moderate (*I*^2^ = 67.6%) [59]. Benthic group as a moderator explained a significant portion of heterogeneity within the data (Q_6_ = 379.459, *p* < 0.001), but significant residual heterogeneity between studies remained unexplained (Q_174_ = 1367.567, *p* < 0.001), justifying further subgroup analysis to investigate causes of variation in the effect of upwelling across studies.

### (c) Subgroup analysis

#### (i) Functional morphology

Categorizing groups as either 'calcifying' or 'fleshy' organisms did not indicate a distinct pattern of positive or negative effect of upwelling on either functional group (*p* = 0.469, *p* = 0.337, respectively) (electronic supplementary material, figure S6).

#### (ii) Depth category

**Figure 2.**
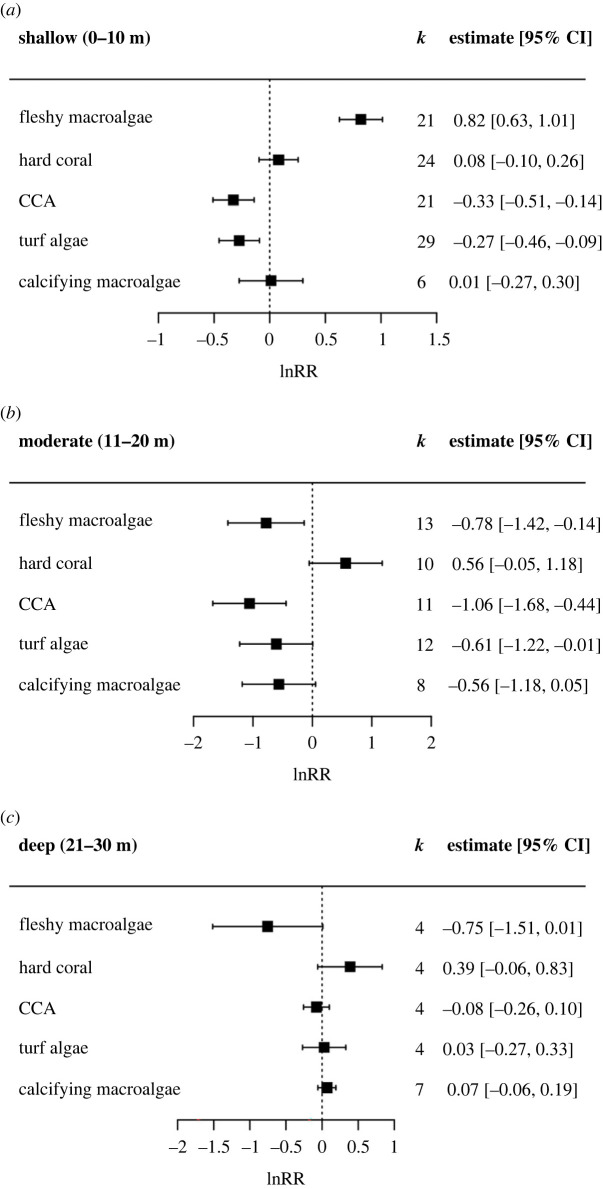
The lnRR (natural logarithm of response ratio) showing the effect of upwelling on the percentage cover of benthic groups separated by depth category: (*a*) shallow, (*b*) moderate and (*c*) deep. Boxes and error bars represent lnRR pooled effect sizes and 95% confidence intervals; values falling to the left of the dotted line indicate a negative effect of upwelling on the percentage cover of benthic groups, and to the right a positive effect. *k* represents the number of case studies that consider each benthic group included in the meta-analysis. Note difference in *x*-axis scales across plots.

Benthic groups within each depth category showed variable responses to upwelling. Notably, upwelling had a significant positive effect on fleshy macroalgae in shallow sites (*p* < 0.001), a significant negative effect in moderate depths (*p* = 0.017), and a visual but non-significant negative effect at deep sites (*p* = 0.053) (figure 2).

#### (iii) Geographic location

**Figure 3.**
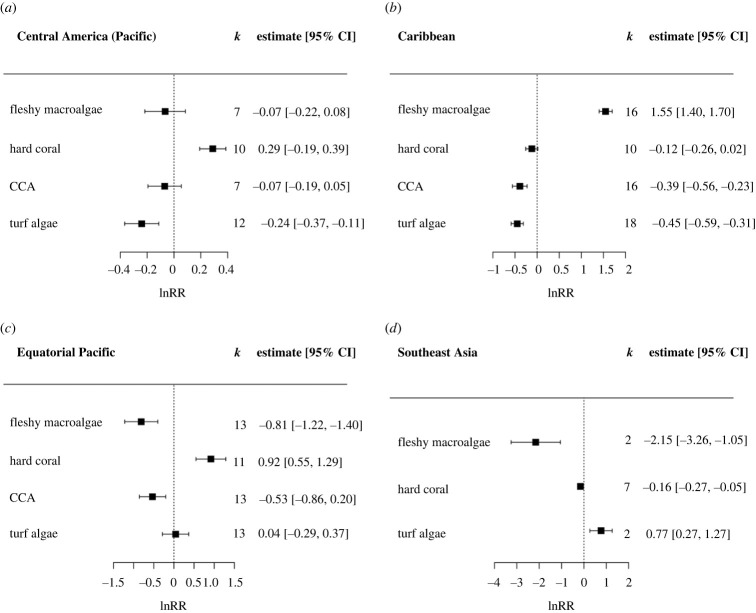
The lnRR (natural logarithm of response ratio) showing the effect of upwelling on the percentage cover of benthic groups separated by location: (*a*) Central America (Pacific), (*b*) Caribbean, (*c*) Equatorial Pacific and (*d*) Southeast Asia. Boxes and error bars represent lnRR pooled effect sizes and 95% confidence intervals; values falling to the left of the dotted line indicate a negative effect of upwelling on the percentage cover of benthic groups, and to the right a positive effect. *k* represents the number of case studies that consider each benthic group included in the meta-analysis. Note difference in *x*-axis scales across plots.

Subgroup analysis of regionally clustered benthic groups was undertaken to explore the variability of upwelling impacts across geographic location. Upwelling in the Caribbean resulted in a significant decrease in turf algae and CCA cover (*p* < 0.001 for both groups). By contrast, fleshy macroalgae showed a mean 371% increase with upwelling in this region (*p* < 0.001). A significant positive effect on hard coral was observed at sampling locations on the Pacific coast of Central America and in the Equatorial Pacific (*p* < 0.001 for both) (figure 3).

#### (iv) Proximity to people

**Figure 4.**
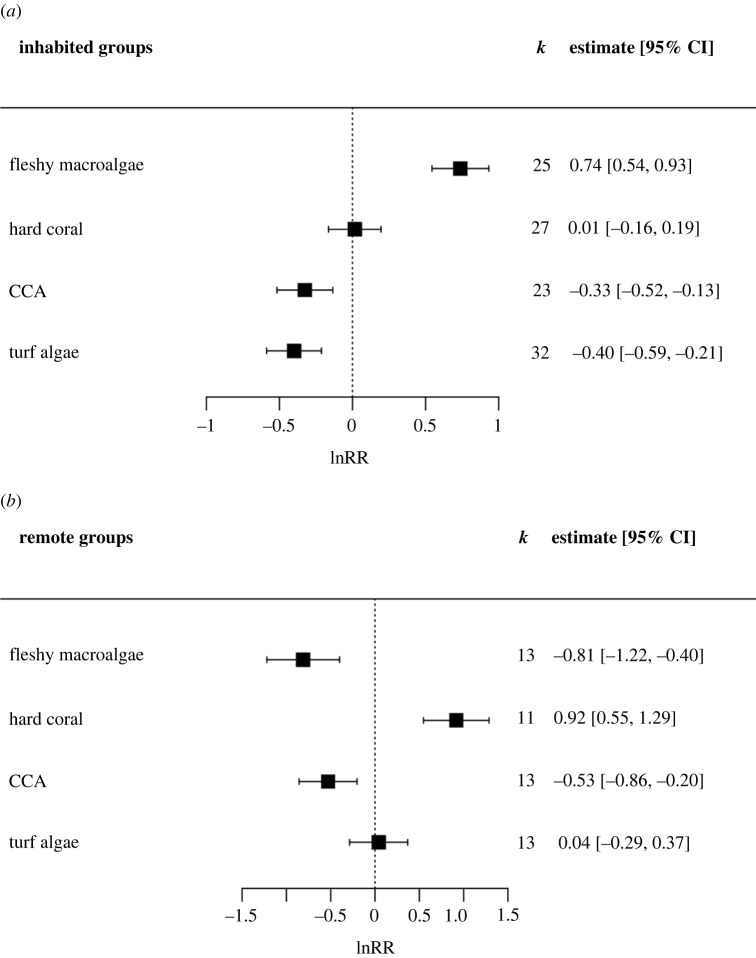
The lnRR (natural logarithm of response ratio) showing the effect of upwelling on the percentage cover of benthic groups separated into *a*) inhabited and *b*) remote locations. Boxes and error bars represent lnRR pooled effect sizes and 95% confidence intervals; values falling to the left of the dotted line indicate a negative effect of upwelling on the percentage cover of benthic groups, and to the right a positive effect. *k* represents the number of case studies that consider each benthic group included in the meta-analysis. Note difference in *x*-axis scales across plots.

When categorized as 'inhabited' or 'remote' and with low validity studies removed, all remaining remote studies were undertaken around Jarvis Island in the Equatorial Pacific. Upwelling resulted in a 110% increase in fleshy macroalgal cover in inhabited locations, but a 56% decrease around Jarvis Island. Upwelling did not have a significant impact on hard coral cover in inhabited areas but coincided with a 150% increase on Jarvis' remote reefs (figure 4).

#### (v) Temperature decrease

Meta-regression showed upwelling intensity, measured as the mean temperature drop, was not a significant predictor of changes in percentage cover of benthic groups (Q_moderator, 153_ = 1701.426, *p* = 0.654). Further subgroup analysis was undertaken to assess the impact of temperature drop on cover of individual groups. A significant negative effect of temperature drop on the percentage cover of hard coral and calcifying macroalgae was detected (Q_moderator, 1_ = 10.959, *p* < 0.001, and Q_moderator, 1_ = 5.546, *p* = 0.019, respectively) (electronic supplementary material, figure S7).

#### (vi) Temporal versus spatial comparison of upwelling

**Figure 5.**
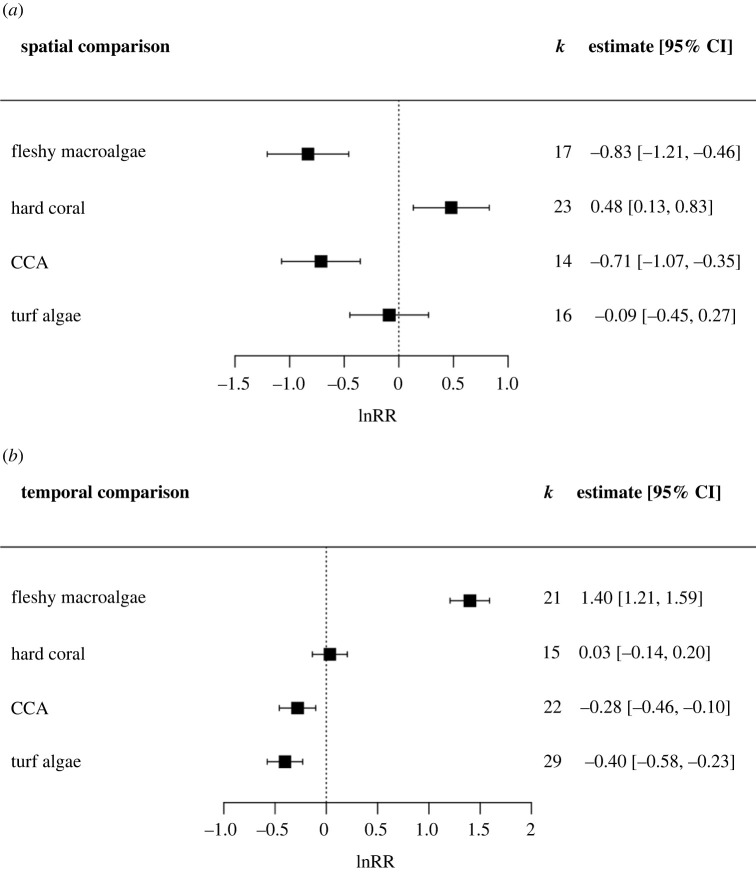
The lnRR (natural logarithm of response ratio) showing the effect of upwelling on the percentage cover of benthic groups impacted by (*a*) spatial and (*b*) temporal (seasonal) upwelling. Boxes and error bars represent lnRR pooled effect sizes and 95% confidence intervals; values falling to the left of the dotted line indicate a negative effect of upwelling on the percentage cover of benthic groups, and to the right a positive effect. *k* represents the number of case studies that consider each benthic group included in the meta-analysis. Note difference in *x*-axis scales across plots.

Fleshy macroalgal cover significantly increased in response to seasonal upwelling (*p* < 0.001), but significantly decreased with spatially distinct upwelling (*p* < 0.001). Hard coral cover was not significantly impacted by seasonal upwelling but significantly increased with spatially distinct upwelling (*p* = 0.007) (figure 5).

## 4. Discussion

The role of upwelling in structuring coral reef benthic communities has not been comprehensively synthesized [10,21,29]. By conducting a systematic review and meta-analysis, we show that upwelling is correlated with significant changes in the percentage cover of benthic groups on coral reefs. Response patterns vary considerably when sub-analysed across geographic location, depth and, most notably, with proximity to human population centres. Responses also vary depending on whether upwelling is seasonally variable.

The pooled effect of upwelling from all studies resulted in an overall increase in fleshy macroalgal cover. This is unsurprising, given that macroalgae are well documented to be opportunistic, able to efficiently utilize heightened water column nutrient concentrations therefore outcompeting slower growing hard coral species [10,63]. This trend was not observed across all geographic locations, however. When sub-analysed by study region, only in the Caribbean did fleshy macroalgal cover respond positively to upwelling. In the Equatorial Pacific (Jarvis Island) and Southeast Asia (Thai Similan Islands), upwelling had a significant negative effect on macroalgal cover. This suggests that upwelling has differential effects on fleshy macroalgae dependant on other extrinsic conditions, such as co-occurring anthropogenic stressors.

While Jarvis Island can be categorized as truly remote, the Thai Similan Islands are moderately free from local human pressure; although subject to heavy dive tourism, the closest population centre is located approximately 60 km away. Our results support the findings of other studies that fleshy macroalgal cover increases in response to upwelling when co-occurring with other anthropogenic stressors, but not in more remote locations [29,30]. The reduction in herbivorous fish abundance with increased fishing pressure that coincides with proximity to human populations is also likely facilitating the positive response of macroalgae to upwelling. On remote reefs where herbivory is high, algal responses to increased nutrient concentrations are moderated by top-down grazing pressure [64]. By contrast, in the Caribbean where over-fishing is recognized as a driver of coral decline [65], upwelling was linked to fleshy macroalgal proliferation in this study. These results are suggestive of differential responses of coral reef communities to upwelling in highly populated areas compared with reefs not subject to direct local human pressures. However, the paucity of evidence linking upwelling with reef communities in remote locations highlights the need for further research to disentangle the effects of gradients in natural and anthropogenic nutrient sources.

The impact of upwelling on fleshy macroalgal cover also varied with depth. Upwelling resulted in an increase in fleshy macroalgal cover in shallow depths, but a decrease in moderate and deeper depths. This may be due to the higher levels of light attenuation at depth, depriving algae of energy for photosynthesis, although this pattern is likely to be species specific [66]. This highlights the need for future studies to identify macroalgal responses to upwelling with higher taxonomic or functional specificity, as different algal species will occupy different ecological niches at varying depths.

Algal assemblages on coral reefs are highly spatially and temporally variable [67], as emphasized by the results of this study. Fleshy macroalgal cover increased by 306% in response to seasonal upwelling, yet a spatial comparison of upwelling and non-upwelling sites correlated with a 56% drop in cover with upwelling. By contrast, hard coral cover was not impacted by seasonal upwelling but increased by 62% in upwelling compared with non-upwelling sites. This can likely be explained by the difference in response times of fleshy macroalgae and hard corals to increases in allochthonous energy resources, although this requires further research [68]. Future studies could focus on quantifying the responses of different benthic groups to gradients in energy availability over different time-scales, particularly organisms such as hard corals that employ a mixotrophic feeding strategy [18].

Although only two studies considered soft coral responses to upwelling, an overall significant positive effect of upwelling on soft coral cover was observed. Soft corals are able to lean more heavily on heterotrophy than scleractinian corals [69]. Given that upwelling can increase plankton abundance resulting from enhanced nutrient concentrations, this offers an explanation for increased soft coral abundance at upwelling exposed sites [70,71].

An overall negative effect of upwelling on CCA abundance was observed, a trend that was also reflected in subgroup analysis by geographic location and remote versus inhabited areas. CCA is an important benthic calcifier on coral reefs, functioning to consolidate reef structure, binding segments of reef and contributing to overall reef accretion [72]. As a biomineralizing group that requires calcium carbonate to form skeletal structure, CCA is highly vulnerable to the deleterious effects of ocean acidification [73]. Upwelling can lower seawater pH, which could be preventing or diminishing CCA growth [74] despite the beneficial increase in available nutrients to the algae.

The effect of upwelling on hard coral cover was highly variable, with an almost even distribution of reported positive and negative responses in coral cover across studies. As expected, hard coral exhibited differential responses to upwelling when separated into remote and inhabited locations. Upwelling resulted in a 144% increase in hard coral cover on the remote reefs surrounding Jarvis Island, but did not have a significant effect on reefs subject to direct human pressures. Williams *et al*. [28] found that on remote reefs in unpopulated areas, background increases in chlorophyll-*a* (a proxy for phytoplankton biomass) coincide with a decrease in macroalgal cover and an increase in hard coral and CCA dominance. This apparent competitive advantage to key calcifying organisms could explain some of the variation in hard coral cover in response to upwelling found in the present study. In essence, the impacts of upwelling on the abundance of hard coral are diminished when local anthropogenic stressors override the natural variation in associated biophysical parameters. The presence of human population centres drowns out natural biophysical relationships by fundamentally changing the environmental conditions within which coral reefs have evolved to thrive. While natural nutrient enrichment driven by upwelling may provide a benefit to corals in terms of growth and productivity, the volume and type of nutrients deposited by anthropogenic activities surpasses the tipping point at which nutrient enrichment triggers negative impacts on coral health [17,75].

The results of this meta-analysis highlight the paucity of evidence linking physical oceanographic processes with coral reef benthic ecology. Just 17 publications directly measured the effects of fluctuations in environmental parameters associated with upwelling with changes in the percentage cover of benthic groups. Study effort was highly spatially clustered, highlighting the need for further research into the impact of upwelling on benthic community structure across scales and geographies. Our ability to develop nuanced and adaptive management strategies for maintaining coral reefs that support high biodiversity and provide key ecosystem services to people requires a thorough understanding of both natural environmental drivers and anthropogenic stressors [3,35,40]. A number of studies have explored the concept of upwelling zones as potential refugia for corals from thermal stress [14,35,76,77]. The present study shows that upwelling may benefit hard corals, demonstrating that upwelling results in an increase in hard coral cover in some (but not all) locations, and particularly where local anthropogenic stressors are lacking.

If thermal refugia are to be included in the arsenal of conservation scientists and reef managers, care must be taken when selecting sites. The protective capacity of upwelling seems to be localized to specific geographic areas and is unlikely to provide a failsafe guard against coral mortality under extreme temperature events. In order for upwelling to confer protection from thermal stress, Chollett *et al*. [77] identified two conditions that must be met; firstly, the thermal stress event and the presence of upwelling must occur synergistically; and secondly, the occurrence of upwelling during the warming event must result in a meaningful decrease in heat stress [77]. In summary, upwelling cannot be considered a panacea to heat stress but may be a useful tool for managers to factor into reef management plans and the distribution of conservation resources.

This study has highlighted the differential impacts of upwelling, varying as a function of both environmental and anthropogenic context [78]. In order to fully understand the interplay between physical oceanographic drivers of change on coral reefs and anthropogenic stressors, further interdisciplinary research joining physical oceanography, benthic ecology and social science is needed to effectively manage coral reefs in the Anthropocene [3,79].

Should such an evidence synthesis exercise be undertaken again in a decade, a more robust and comprehensive understanding of the interplay between upwelling and benthic community structure could be obtained. Future research should aim for more detailed quantification of upwelling parameters, including changes in *in situ* water column nutrient concentrations during upwelling events. Further, by identifying species within benthic groups to a higher taxonomic resolution, the variability in responses of individual species could be explored, particularly algae which perhaps do not fall neatly into ‘fleshy macroalgae’ and ‘calcifying macroalgae’. And finally, developing manipulative experiments that seek to separate the synergistic impacts of temperature drop and nutrient increase associated with upwelling events would allow greater understanding of the mechanisms driving benthic community structure. If these aims are met, such research may provide further clarity to decision makers on the impacts of natural oceanographic forces on coral reefs, so that these may be taken into consideration when managing anthropogenic stressors and selecting reefs for focused conservation efforts. Reef management that does not account for natural variation and environmental drivers of change is limited by a lack of understanding of environmental context and natural carrying capacity of the reefs they are trying to preserve [6]. The results of this review can be used by policy and decision makers when determining spatial bounds for reef management, aiding optimal resource allocation and informed reef conservation policy that accounts for the impacts of environmental variation.

## Data Availability

Data can be obtained from Dryad Digital Repository: https://doi.org/10.5061/dryad.w0vt4b8wg [56]; additional information and R script are available as electronic supplementary material [80].
